# Reliability and validity of the Japanese short-form arthritis self-efficacy scale in patients with knee osteoarthritis: A cross-sectional study

**DOI:** 10.1371/journal.pone.0292426

**Published:** 2023-10-20

**Authors:** Daisuke Uritani, Takanari Kubo, Yuuka Yasuura, Tadashi Fujii

**Affiliations:** 1 Faculty of Health Science, Department of Physical Therapy, Kio University, Kitakasturagigun, Nara, Japan; 2 Department of Physical Therapy, Osaka Kawasaki Rehabilitation University, Kaizuka, city, Osaka, Japan; 3 Department of Rehabilitation, Shimada Hospital, Habikino city, Osaka, Japan; 4 Department of Orthopaedic Surgery, Kashiba Asahigaoka Hospital, Kashiba city, Nara, Japan; Mugla Sitki Kocman Universitesi, TURKEY

## Abstract

Self-efficacy is the belief that one can perform a specific behavior or task in the future, and it has been associated with physical and psychological aspects in people with chronic musculoskeletal disorders. The self-efficacy of individuals with arthritis can be assessed using the Arthritis Self-Efficacy Scale. The 8-item Short-Form ASES (ASES-8) has been employed in recent times. However, the reliability and validity of the Japanese ASES-8 (ASES-8J) have not been investigated. Therefore, this study aimed to investigate the reliability and validity of the ASES-8J. Overall, 179 Japanese participants with knee osteoarthritis (OA) were enrolled. Cronbach’s alpha was calculated to confirm internal validity. Intraclass correlation coefficients (ICCs) were used to estimate test-retest reliability. Construct validity was analyzed using the Pain Self-Efficacy Questionnaire (PSEQ) and the problem-solving and positive thinking subscales of Brief Coping Orientation to Problems Experienced (Brief COPE). Discriminant validity was analyzed by comparing “worse” and “better” groups based on pain severity; short-form version of Depression, Anxiety, and Stress Scale-21 (DASS-21); Brief Fear of Movement Scale for Osteoarthritis (BFOMSO); Pain Catastrophizing Scale (PCS); and physical function subscale of Western Ontario and McMaster Arthritis Index. Cronbach’s alpha and ICC were 0.94 and 0.81, respectively. Correlation coefficients among ASES-8J, PSEQ, and Brief COPE problem-solving and positive thinking subscales were 0.42, 0.43, and 0.32, respectively. Regarding the depression and stress subscales of DASS-21, BFOMSO, and PCS, the worse group showed significantly lower ASES-8J scores than the better group. Coefficients of correlation among ASES-8J, PSEQ, and the problem-solving and positive thinking subscales of Brief COPE were low to moderate. These findings suggest that the ASES-8J is a valid and reliable tool for assessing self-efficacy in Japanese patients with knee OA and can facilitate comparisons of arthritis self-efficacy between Japanese patients and non-Japanese patients.

## Introduction

Self-efficacy is the belief that one can perform a specific behavior or task in the future [1], and it has been associated with physical and psychological aspects in people with chronic musculoskeletal disorders. Higher self-efficacy is related to higher physical function, quality of life, work efficiency, and patient satisfaction in chronic musculoskeletal disorders [2].

The self-efficacy of individuals with arthritis can be assessed using the Arthritis Self-Efficacy Scale (ASES) [3]. Originally, the ASES comprised the following 20 items: five, nine, and six items of self-efficacy for pain, physical function, and other symptoms, respectively [3]. It has been validated as a reliable measure of self-efficacy in patients with knee osteoarthritis (OA) [3]. Additionally, the 8-item Short-Form ASES (ASES-8) [4] has recently been employed because it is significantly less burdensome for patients than the original ASES. The ASES-8 comprises two ASES pain subscale items, four ASES of other symptom subscale items, and two new items related to preventing fatigue and pain from interfering with daily activities [4]. Moreover, the ASES-8 presented good validity and reliability [5,6]. Accordingly, it has been translated into several languages [7–11], and all have demonstrated good reliability and validity.

Self-efficacy is also related to physical and psychological aspects in people with knee OA. Particularly, self-efficacy is associated with the severity of knee pain in patients with knee OA [12]. Furthermore, self-efficacy affects the prognosis of patients with knee OA who have undergone total knee arthroplasty [13]. Moreover, self-management education can enhance the self-efficacy of patients with knee OA [14]. Therefore, self-efficacy is an important treatment target for improving physical function [15] and the self-management of symptoms [16,17] in patients with knee OA.

Knee OA is a crucial public health issue in Japan. Its prevalence in Japan is significantly higher than that in the United States and Europe [18]. Although there are many opportunities to treat individuals with knee OA, their physical and psychosocial aspects, including self-efficacy, should be evaluated. However, no Japanese version of the ASES-8 (ASES-8J) exists. Accordingly, we translated the original English version of the ASES-8 into Japanese with permission from the original developer (K. Lorig) based on the development process described by Beaton et al. [19] Based on a pilot test that evaluated the Japanese cultural adaptations, we developed the final version of the ASES-8J [20]. However, its reliability and validity have not been investigated. Therefore, this study aimed to determine the reliability and validity of the ASES-8J.

## Materials and methods

### Ethics statements

The present study adhered to Good Clinical Practice guidelines, as outlined by the International Conference on Harmonisation. Furthermore, this study was conducted in accordance with the principles of the Declaration of Helsinki and was approved by the Research Ethics Committees of Kio University (approval no.: H29-08) and Kashiba Asahigaoka Hospital (approval no.: 2018111002). All participants provided written informed consent.

### Participants

The participants were Japanese patients with painful knee OA recruited from five clinics and hospitals in different regions of Japan. Participants were recruited from December 5, 2018 to July 16, 2021. Inclusion criteria were age >45 years, Kellgren–Lawrence grade ≥2 [21], and knee pain on most days of the past month. Exclusion criteria were a history of knee joint replacement on the knee being studied or any other condition affecting lower limb function to a greater extent than knee pain. Patients suspected of cognitive decline in interviews or verbal communication before requesting research cooperation were also excluded, although no specific screening tests were conducted. Additionally, the most symptomatic knees were selected for investigation from patients in whom both knees were symptomatic. Sample size was determined following a general recommendation that an adequate sample size should include >100 participants [22] and that at least 50 participants must be included to confirm test-retest reliability [23].

### Outcome measures

#### Arthritis self-efficacy

Arthritis self-efficacy was measured using the ASES-8J [20], which comprises eight items that assess self-efficacy for arthritic pain and other symptoms. Responses range from 1 (very uncertain) to 10 (very certain). The scale score is the mean of eight items, where higher scores indicate greater levels of arthritis self-efficacy.

The ASES-8J was developed based on the translation process described by Beaton et al. [19]. As the first step, forward translation was performed by translating the original version of the ASES-8 into Japanese by two Japanese individuals who were bilingual in Japanese and English. One translator was a physical therapist working at an educational and research institution, whereas the other was a university employee with no medical background. Forward translation was independently performed by two translators. In the second stage, a physical therapist working at an educational and research institution participated as a coordinator, and the two forward translators along with the coordinator merged their translations. In the third stage, the forward translations integrated in the second stage were translated back into English by two Japanese individuals who were bilingual in Japanese and English. These back translators were nurses working at educational and research institutions. Back translation was independently performed by two back translators without referring to the original version. In the fourth stage, the two back translators and the coordinator discussed and merged the two back translations. Then, the two forward translators also joined in the process, and the integrated back translation was examined to determine whether it was conceptually equivalent to the original version, and the final reverse translation was completed. In the fifth step, we asked the original author to confirm the back translation and examine whether the English expressions in the back translation differed from the original author’s intent. The back translation was revised as necessary to create the final version of the ASES-8J.

The sufficiency of the linguistic and face validity of the ASES-8J has been confirmed [20]. Furthermore, the high internal consistency of the ASES-8J and the absence of ceiling or floor effects in the pilot test were confirmed [20]. Participants completed the ASES-8J again within 2 weeks, which was the next visit from the first assessment of the ASES 8-J.

### Pain intensity

Pain intensity was assessed using a 10-point numeric rating scale (0 = no pain and 10 = worst pain possible) [24] for average knee pain during the past week. Such measurements have demonstrated reliability in OA [25].

#### Pain self-efficacy

Pain self-efficacy was measured using the Pain Self-Efficacy Questionnaire (PSEQ) [26]. The PSEQ is an established 10-item measure of pain self-efficacy that is adopted in clinical and research settings. Responses range from zero (not at all confident) to six (completely confident), with higher scores indicating greater pain self-efficacy. Notably, the Japanese version of the PSEQ (PSEQ-J) has been validated [27].

#### Coping strategy

Coping strategy was measured using the Short-Form Coping Orientation to Problems Experienced (Brief COPE) [28]. The Brief COPE comprises four strategies: seeking social support, problem-solving, avoidance, and positive thinking [29]. Responses range from 1 to 4, where higher scores indicate a greater likelihood that the difficulty will be addressed by the strategy stated in the question. The Brief COPE was also translated into Japanese and validated [30]. We calculated the subtotal scores of the problem-solving and positive thinking subscales.

#### Depression, anxiety, and stress

Depression, anxiety, and stress were measured via the short-form version of the Depression, Anxiety, and Stress Scale-21 (DASS-21) [31]. The DASS-21 [32] comprises seven items for three subscales (depression, anxiety, and stress). Responses range from 0 (did not apply to me) to 3 (apply to me very much or most of the time). Additionally, scores from each subscale were summed and multiplied by two to obtain a subscale score ranging from 0 to 42, where higher scores indicate a greater level of symptoms. Notably, the English version has high internal consistency and construct validity [31,32]. Although the Japanese version of the DASS [31] was used for participants in Japan, the reliability and validity of the translated version are yet to be reported.

#### Fear of movement

Fear of movement was assessed using the Brief Fear of Movement Scale for Osteoarthritis (BFOMSO) [33], which comprises six items extracted from the Tampa Scale for Kinesiophobia (TSK) [34], using a 4-point scale from “strongly agree” to “strongly disagree” to assess the fear of injury or re-injury because of movement with values ranging from 6 to 24. Higher scores indicate a greater fear of movement. The same six questions from the BFOMSO [33] were extracted from the Japanese version of the TSK [35]. Additionally, the original version of the TSK has been translated into Japanese and linguistically validated [35]. The Japanese version of the TSK is psychometrically reliable and valid for fear of movement detection in the Japanese population with neck-to-back pain [36].

#### Pain catastrophizing

Pain catastrophizing was evaluated using the Pain Catastrophizing Scale (PCS) [37] that comprises 13 items that assess tendencies to ruminate, feel helpless about pain, and magnify pain on a scale of 0–4. The total score ranges from 0 to 52 (subscale of rumination: 0–16, helplessness: 0–24, magnification: 0–12), with higher scores indicating greater pain catastrophizing. Furthermore, it has high internal consistency and is associated with heightened pain, psychological distress, and physical disability among adults [38]. Therefore, the Japanese version of the PCS [39] was employed. Moreover, the reliability and validity of the Japanese PCS version have been confirmed as acceptable [39].

#### Physical function

Physical function was assessed using the Japanese versions of the physical function subscale of the Western Ontario and McMaster Universities Osteoarthritis Index (WOMAC) Likert version [40]. The physical function subscale of the WOMAC has 17 questions, with five response options ranging from 0 (indicating no physical dysfunction) to 4 (indicating extreme physical dysfunction), where higher scores indicate a greater level of physical dysfunction. The Japanese version of the WOMAC was considered reliable, valid, and responsive for assessing the effectiveness of total knee arthroplasty in the Japanese context despite cultural differences from Western countries [41].

#### Patients’ subjective change in pathological condition

Subjective changes under pathological conditions were measured using the Patient Global Impression of Change (PGIC) [42]. The PGIC is a 7-point categorical scale, with responses ranging from 1 (very much improved) to 7 (very much worse), and it is used to evaluate a patient’s subjective change in condition. The value four indicates no change. The PGIC was used to determine patients’ subjective changes in their pathological condition of knee OA status between the first and second measurements of the ASES-8J. Therefore, the PGIC was measured simultaneously with the second measurement ASES-8J.

### Data analysis

Descriptive statistics were calculated for all variables to summarize participant characteristics. Categorical data are presented as frequencies and percentages. Furthermore, the study variables are presented as mean and standard deviation (SD) for normally distributed variables and as median and interquartile range for non-normally distributed variables. Absolute measurement errors were estimated by the standard error of measurement (SEM) and by converting the SEM into the minimal detectable change (MDC) at a 95% confidence interval (CI) (MDC_95_) (MDC_95_ = 1.96 × √2 × SEM) [23].

The test-retest reliability of the questionnaire was calculated using the intraclass correlation coefficient (ICC) for participants who experienced no change in their pathological condition (i.e., stability) between the first and second measurements of the ASES 8-J based on the PGIC (PGIC = 4). An ICC of >0.75 indicates excellent reliability [43]. Additionally, Cronbach’s alpha was estimated to confirm internal consistency, where a Cronbach’s alpha of ≥0.7 is considered acceptable internal consistency as a psychometric assessment tool [44]. Internal consistency was assessed by calculating the corrected item-total scale correlation. A value of ≥0.4 was considered an adequate item for internal consistency. Moreover, floor and ceiling effects were confirmed based on the data distribution. If ≥15% of responders presented a minimum or maximum score, floor or ceiling effects were identified [23].

Correlations between the ASES-8J, PSEQ, and problem-solving and positive thinking subscales of the Brief COPE were calculated for concurrent validity using correlation coefficients (Pearson or Spearman, according to statistical distribution). The correlation coefficients were categorized as follows: 0.0–0.2, 0.2–0.4, 0.4–0.6, 0.6–0.8, and 0.8–1.0, indicating very weak, weak, moderate, strong, and very strong relationships, respectively [45]. Furthermore, exploratory factor analysis was performed to evaluate the construct validity of the ASES-8J, where a loading factor of >0.4 was the cut-off point for item retention. Therefore, Kaiser–Meyer–Olkin (KMO) values and Bartlett’s test sphericity were used to assess the suitability of the factor analysis.

Additionally, the ASES-8J was compared between the two groups (low- vs. high-score groups) to evaluate discriminant validity based on pain severity, depression, anxiety, stress, fear of movement, and pain catastrophizing using the unpaired t-test. First, pain severity was categorized into mild and moderate/severe (numerical rating scale <4/10 vs. ≥4/10) [46]. Second, depression, anxiety, and stress were classified into normal and mild to extremely severe (depression subscale of the DASS-21 <10/21 vs. ≥10/21, anxiety subscale of the DASS-21 <8/21 vs. ≥8/21, and stress subscale of the DASS-21 <15/42 vs. ≥15/42) [47]. Based on previous studies [34,48], the cut-off value of the original TSK was set at 40/68 points (58.8%). Therefore, the cut-off value of the BFOMSO was 58.8% of the total score, which was 14/24. Finally, pain catastrophizing was categorized into two groups based on a previously reported cut-off value (PCS <24/52 vs. PCS ≥24/52) [49].

Statistical analyses were performed using Statistical Package for Social Sciences software version, 22.0 (IBM Corp., Armonk, NY, USA). Statistical significance was set at *p*<0.05.

## Results

Data were collected from September 11, 2018, to October 29, 2021. Overall, 226 participants were recruited. Among them, 12 were excluded because of missing data in the questionnaire and 35 because they did not meet the inclusion criteria, including age, KL grade, and pain severity. Finally, 179 individuals (52 male and 127 female participants; mean age: 70.1±8.9 years) with knee OA were included. The mean, SD, and SEM ASES-8J scores were 5.7, 2.2, and 0.16, respectively, and MDC_95_ was 0.44. Table 1 summarizes participant characteristics.

**Table 1:** Participants’ characteristics.

Patient characteristics	Number	Mean (SD)
Age (years)	179	70.1 (8.9)
Sex		
Female	127	
Male	57	
Height (cm)	179	158.1 (7.8)
Weight (kg)	179	61.8 (12.1)
Body mass index (kg/m^2^)	179	24.6 (3.9)
Employment status		
Employed	56	
Unemployed/Retired	114	
Missing data	9	
Kellgren–Lawrence grade		
Ⅱ	60	
Ⅲ	67	
Ⅳ	52	

SD: standard deviation.

### Reliability

The Cronbach’s alpha was 0.94, indicating high internal consistency. The corrected item-total correlations ranged from 0.66 to 0.87. Notably, the alpha value remained high (0.93–0.94) if single items were deleted (Table 2).

**Table 2:** Results of outcome measures.

Patient characteristics	Number	Mean (SD)/median (Q1–Q3)
Self-efficacy (ASES-8J)	179	5.7 (2.2)
Pain (NRS)	179	5.0 (2.2)
Pain self-efficacy (PSEQ)	122	44.5 (33.75–50.00)
Coping (Brief COPE)	122	
Problem-solving		11.7 (2.7)
Positive thinking		15.0 (3.8)
Depression (DASS-21)	122	4.0 (0.0–10.0)
Anxiety (DASS-21)	122	2.0 (0.0–4.5)
Stress (DASS-21)	122	4.0 (0.0–10.0)
Physical function (WOMAC)	122	11.0 (6.00–18.00)
Fear of movement (BFOMSO)	115	12.0 (9.00–15.00)
Pain catastrophizing (PCS)	115	20.0 (12.00–28.00)

ASES: Arthritis Self-Efficacy Scale; BFOMSO: Brief Fear of Movement Scale for Osteoarthritis; COPE: Coping Orientation to Problems Experienced; DASS-21: Depression, Anxiety, and Stress Scale-21; NRS: Numerical rating scale; PCS: Pain Catastrophizing Scale; PSEQ: Pain Self-Efficacy Questionnaire; SD: Standard deviation; WOMAC: Western Ontario and McMaster Universities Osteoarthritis Index; Q: Quartile.

Fifty-four participants with a PGIC score of 4 were included in determining test-retest reliability. The mean score of the first and second ASES-8J measurements was 5.7±2.0. ICC (95% CI) was 0.81 (0.69–0.99).

Additionally, five participants (2.8%) presented a minimum score (1.0), and one participant (0.6%) showed a maximum score (10.0). Therefore, no floor or ceiling effects were observed.

### Validity

The KMO value was 0.90, and Bartlett’s spherical test was significant (*p* < 0.01 in exploratory factor analysis). These values indicate that factor analysis was feasible. Furthermore, the results showed that all eight items were loaded on a single factor for the full sample and explained 71.09% of the variance. Moreover, factor loadings for each item ranged from 0.68 to 0.90 (Table 3).

**Table 3:** Factor loadings and item performance of the ASES-8J.

Item	Factor loading	Corrected item-total correlation	Cronbach’s alpha if the item was deleted
1	0.68	0.66	0.94
2	0.75	0.74	0.94
3	0.83	0.83	0.93
4	0.83	0.80	0.93
5	0.89	0.87	0.93
6	0.79	0.75	0.94
7	0.89	0.84	0.93
8	0.90	0.83	0.93

ASES: Arthritis Self-Efficacy Scale.

The ASES-8J had moderate to weak significant correlations with the PSEQ (*ρ* = 0.42) and problem-solving (*r* = 0.43) and positive thinking (*r* = 0.32) subscales of the Brief COPE (Table 4).

**Table 4:** Correlation between ASES-8J and psychological outcomes.

	PSEQ	Problem-solving of Brief COPE	Positive thinking of Brief COPE
ASES-8J	0.42**	0.43**	0.32**

**p < 0.01.

ASES: Arthritis Self-Efficacy Scale; COPE: Coping Orientation to Problems Experienced; PSEQ: Pain Self-Efficacy Questionnaire.

Regarding discriminant validity, the higher depression group had a lower ASES-8J score than the lower depression group (mean difference [95% CI]: -1.31 [-2.20, -0.42]). The higher stress group also had a lower ASES-8J score than the lower stress group (mean difference [95% CI]: -1.36 [-2.47, -0.25]). The higher fear of movement and pain catastrophizing groups presented lower ASES-8J scores than the lower fear of movement and pain catastrophizing groups (mean difference [95% CI]: -1.23 [-2.06, -0.39] and -0.89 [-1.73, -0.06], respectively). There were no significant differences in ASES-8J scores between the high- and low-score groups with respect to pain, anxiety, or WOMAC physical function (Table 5).

**Table 5:** Differences in ASES-8J scores between low- and high-score groups.

	Low score	High score	*p*-value	Mean difference (95% CI)
Pain severity (NRS)	<4 (*n* = 52)	≥4 (*n* = 127)		
	5.9±2.3	5.7±2.1	0.55	-0.22 (-0.93, 0.50)
Depression (DASS-21)	<10 (*n* = 90)	≥10 (*n* = 32)		
	6.2±2.2	4.8±2.0	0.004	-1.31 (-2.20, -0.42)
Anxiety (DASS-21)	<8 (*n* = 98)	≥8 (*n* = 24)		
	5.9±2.3	5.5±2.0	0.41	-0.42 (-1.44, 0.59)
Stress (DASS-21)	<15 (*n* = 104)	≥15 (*n* = 18)		
	6.0±2.2	4.7±1.9	0.02	-1.36 (-2.47, -0.25)
Fear of movement (BFOMSO)	<14 (*n* = 71)	≥14 (*n* = 44)		
	6.2±2.3	5.0±2.1	0.004	-1.23 (-2.06, -0.39)
Pain catastrophizing (PCS)	<24 (*n* = 66)	≥24 (*n* = 49)		
	6.1±2.4	5.3±2.0	0.04	-0.89 (-1.73, -0.06)
Physical function (WOMAC)	<21 (*n* = 94)	≥21 (*n* = 28)		
	6.0±2.3	5.2±2.0	0.12	-0.74 (-1.63, 0.14)

ASES: Arthritis Self-Efficacy Scale; BFOM: Brief Fear of Movement Scale for Osteoarthritis; DASS-21: Depression, Anxiety, and Stress Scale-21; NRS: Numerical rating scale; PCS: Pain Catastrophizing Scale; WOMAC: Western Ontario and McMaster Universities Osteoarthritis Index; CI: Confidence interval.

## Discussion

To our knowledge, this is the first study to assess the reliability and validity of the ASES-8J. Herein, the ASES-8J showed acceptable reliability and validity for evaluating arthritis self-efficacy in Japanese patients with knee OA.

Although this study included more women than men, it reflects the general knee population with OA in Japan, which can be explained by the higher prevalence of knee OA among women [18]. Furthermore, the mean ASES-8J score (5.7±2.2) was similar to those of the Spanish version for patients with arthritis (5.9±2.1) [7], a German version for those with rheumatoid arthritis (RA) (5.4±1.6) [8], an Arabic version for those with RA (5.5±1.7), and a Brazilian Portuguese version for those with RA (5.9±2.1).

Cronbach’s alpha for the ASES-8J was 0.94, which is higher than that for the English (0.89) [5], Spanish (0.92) [7], German (0.90) [8], and Arabic (0.88) versions [11]. When Cronbach’s alpha was 0.7, the psychometric measure was deemed as having acceptable reliability [44]. Therefore, the ASES-8J was determined to have acceptable reliability. However, when any one item was removed, Cronbach’s alpha values remained similar or decreased. This result means that each item uniquely contributed to the overall conceptual framework of the ASES-8J. Furthermore, the corrected item-total correlation was high (0.66–0.87), implying that the individual items fit the overall scale appropriately.

Additionally, the ICC of the ASES-8J (0.81–1.0) is considered “almost perfect” [45]. Thus, the ASES-8J demonstrated high test-retest reliability since the ICC of the ASES-8J was 0.81 in this study, indicating that it is stable and has good stability after repeated administration.

The ASES-8 comprises two items from the ASES pain subscale, four items from the ASES other symptoms subscale, and two new items related to the prevention of pain and fatigue that interfere with daily activities. However, because the ASES-8 does not evaluate subscales, the ASES-8J was treated as a one-factor structure. The exploratory factor analysis in this study indicated that the ASES-8J comprised a one-factor structure consistent with previous studies on the other language versions of the ASES-8 [5,8,9]. Therefore, the factor loading values for each item (0.68–0.90) demonstrated that they were good indicators of a single factor.

The ASES-8J had significant correlations with self-efficacy for pain and coping strategies to solve the problem during the assessment of construct validity. However, the correlation between the ASES-8J and PSEQ (*r* = 0.38) and the problem-solving subscale of the Brief COPE (*r* = 0.43) was low to moderate. The ASES-8J includes items about self-efficacy for pain management and fatigue, physical activity, and mood, whereas the PSEQ includes items specific to pain management. Similarly, Brief COPE broadly addresses how to deal with problems in daily life rather than specific events, whereas the ASES-8J is a disease-specific questionnaire. Therefore, the correlation among the ASES-8J, PSEQ, and Brief COPE was weak.

Pain severity and physical function were not associated with ASES-8J scores regarding discriminant validity. Most studies have described a significant association between ASES-8 scores and pain severity [5,8,9,11], although a few previous studies have described the opposite [6,10]. These studies included patients with RA, fibromyalgia, or diverse samples, whereas the current study included only patients with knee OA. Therefore, this may explain why the association between pain severity and ASES-8 scores herein differed from the association reported in previous studies [5,8,9,11]. Additionally, Muller et al. [8] reported that some patients with RA had highly effective coping mechanisms irrespective of pain severity; however, those with low pain levels had little control over their symptoms. Therefore, this study’s population with knee OA might also show a similar trend.

Functional state has been associated with ASES-8 scores [5,6,9–11]. However, different measures have been used to assess the functional aspects of these studies. Therefore, in addition to sample characteristics, this may be why the association between functional state and ASES-8 scores in this study differed from those of previous studies.

Psychological states were also associated with the ASES-8J. Regarding depression, stress, fear of movement, and pain catastrophizing, the high-score (worse) group had lower arthritis self-efficacy than did the low-score (better) group. Additionally, depression showed a significantly negative correlation with the ASES-8 scores in previous studies [5,6,8–11]. In another study, pain catastrophizing was significantly correlated negatively with arthritis self-efficacy using the 20-item ASES [50]. Therefore, in Japanese patients with knee OA, similar to the findings from other countries, arthritis self-efficacy is associated with the psychological characteristics of individuals, irrespective of pain severity and functional state.

This study has some limitations. First, the participants were only Japanese individuals with knee OA. Therefore, further research is needed to evaluate the validity and reliability of the ASES-8J in Japanese patients with different arthritic diseases. Second, this was a cross-sectional study; thus, additional longitudinal studies should investigate the sensitivity of the ASES-8J.

In conclusion, the study results showed acceptable levels of validity and reliability of the ASES-8J among Japanese patients with knee OA, indicating that the ASES-8J is a useful tool for assessing arthritis self-efficacy in Japanese patients with knee OA. Therefore, our results support the greater application of the ASES-8J in clinical and research practices that include Japanese patients with knee OA. Additionally, the ASES-8J can compare arthritis self-efficacy between Japanese patients and those from other countries.

## Supporting information

S1 Dataset (XLSX)

## References

[1] Bandura A. Self-efficacy: toward a unifying theory of behavioral change. Psychol Rev. 1977;84: 191–215. doi: 10.1037//0033-295x.84.2.191 847061

[2] Martinez-Calderon J, Zamora-Campos C, Navarro-Ledesma S, Luque-Suarez A. The role of self-efficacy on the prognosis of chronic musculoskeletal pain: a systematic review. J Pain. 2018;19: 10–34. doi: 10.1016/j.jpain.2017.08.008 28939015

[3] Lorig K, Chastain RL, Ung E, Shoor S, Holman HR. Development and evaluation of a scale to measure perceived self-efficacy in people with arthritis. Arthritis Rheum. 1989;32: 37–44. doi: 10.1002/anr.1780320107 2912463

[4] Patient S, Education Research Center. Arthritis self-efficacy scale. 2022. Available from: http://patienteducation.stanford.edu/research/searthritis.html.

[5] Wilcox S, Schoffman DE, Dowda M, Sharpe PA. Psychometric properties of the 8-item English arthritis self-efficacy scale in a diverse sample. Arthritis. 2014;2014: 385256. doi: 10.1155/2014/385256 25215233 PMC4158258

[6] Turner JA, Ersek M, Kemp C. Self-efficacy for managing pain is associated with disability, depression, and pain coping among retirement community residents with chronic pain. J Pain. 2005;6: 471–479. doi: 10.1016/j.jpain.2005.02.011 15993826

[7] González VM, Stewart A, Ritter PL, Lorig K. Translation and validation of arthritis outcome measures into Spanish. Arthritis Rheum. 1995;38: 1429–1446. doi: 10.1002/art.1780381010 7575693

[8] Mueller A, Hartmann M, Mueller K, Eich W. Validation of the arthritis self-efficacy short-form scale in German fibromyalgia patients. Eur J Pain. 2003;7: 163–171. doi: 10.1016/S1090-3801(02)00097-6 12600798

[9] Gao L, Zhang XC, Li MM, Yuan JQ, Cui XJ, Shi BX. Psychometric properties of the Chinese version of Arthritis self-efficacy Scale-8 (ASES-8) in a rheumatoid arthritis population. Rheumatol Int. 2017;37: 751–756. doi: 10.1007/s00296-016-3640-y 28063069

[10] Silva RVTD, Silva FC, Meireles SM, Natour J. Translation to Brazilian Portuguese, cultural adaptation and psychometric properties of 8-item Arthritis self-efficacy Scale (ASES-8). Sao Paulo Med J. 2019;137: 6–12. doi: 10.1590/1516-3180.2018.0354071218 31116272 PMC9721212

[11] Arab Alkabeya H, Daibes J, Hughes AM, Adams J. The Arabic Arthritis self-efficacy Scale-8 (ASES-8): a valid and reliable measure of evaluating self-efficacy in Palestinian patients with rheumatoid arthritis. Disabil Rehabil. 2021;43: 3827–3833. doi: 10.1080/09638288.2020.1748730 32285716

[12] Urquhart DM, Phyomaung PP, Dubowitz J, Fernando S, Wluka AE, Raajmaakers P, et al. Are cognitive and behavioural factors associated with knee pain? A systematic review. Semin Arthritis Rheum. 2015;44: 445–455. doi: 10.1016/j.semarthrit.2014.07.005 25151034

[13] Magklara E, Burton CR, Morrison V. Does self-efficacy influence recovery and well-being in osteoarthritis patients undergoing joint replacement? A systematic review. Clin Rehabil. 2014;28: 835–846. doi: 10.1177/0269215514527843 24668361

[14] Brand E, Nyland J, Henzman C, McGinnis M. Arthritis self-efficacy scale scores in knee osteoarthritis: a systematic review and meta-analysis comparing arthritis self-management education with or without exercise. J Orthop Sports Phys Ther. 2013;43: 895–910. doi: 10.2519/jospt.2013.4471 24175602

[15] McKnight PE, Afram A, Kashdan TB, Kasle S, Zautra A. Coping self-efficacy as a mediator between catastrophizing and physical functioning: treatment target selection in an osteoarthritis sample. J Behav Med. 2010;33: 239–249. doi: 10.1007/s10865-010-9252-1 20177766

[16] Harrison AL. The influence of pathology, pain, balance, and self-efficacy on function in women with osteoarthritis of the knee. Phys Ther. 2004;84: 822–831. 15330695

[17] Maly MR, Costigan PA, Olney SJ. Determinants of self efficacy for physical tasks in people with knee osteoarthritis. Arthritis Rheum. 2006;55: 94–101. doi: 10.1002/art.21701 16463419

[18] Muraki S, Oka H, Akune T, Mabuchi A, En-yo Y, Yoshida M, et al. Prevalence of radiographic knee osteoarthritis and its association with knee pain in the elderly of Japanese population-based cohorts: the ROAD study. Osteoarthritis Cartilage. 2009;17: 1137–1143. doi: 10.1016/j.joca.2009.04.005 19410032

[19] Beaton DE, Bombardier C, Guillemin F, Ferraz MB. Guidelines for the process of cross-cultural adaptation of self-report measures. Spine (Phila Pa 1976). 2000;25: 3186–3191. doi: 10.1097/00007632-200012150-00014 11124735

[20] Uritani D, Imagita H, Fukumoto T, Horiuchi M, Shimizu S. Development of an 8-item Japanese Arthritis self-efficacy Scale (ASES-8J) and investigation of its usefulness in patients with osteoarthritis of the knee. Jpn J Rehabil Med. 2020;57: 79–85.

[21] Kellgren JH, Lawrence JS. Radiological assessment of osteo-arthrosis. Ann Rheum Dis. 1957;16: 494–502. doi: 10.1136/ard.16.4.494 13498604 PMC1006995

[22] Terwee CB, Mokkink LB, Knol DL, Ostelo RW, Bouter LM, de Vet HC. Rating the methodological quality in systematic reviews of studies on measurement properties: a scoring system for the COSMIN checklist. Qual Life Res. 2012;21: 651–657. doi: 10.1007/s11136-011-9960-1 21732199 PMC3323819

[23] Terwee CB, Bot SD, de Boer MR, van der Windt DA, Knol DL, Dekker J, et al. Quality criteria were proposed for measurement properties of health status questionnaires. J Clin Epidemiol. 2007;60: 34–42. doi: 10.1016/j.jclinepi.2006.03.012 17161752

[24] Williamson A, Hoggart B. Pain: a review of three commonly used pain rating scales. J Clin Nurs. 2005;14: 798–804. doi: 10.1111/j.1365-2702.2005.01121.x 16000093

[25] Bellamy N. Osteoarthritis clinical trials: candidate variables and clinimetric properties. J Rheumatol. 1997;24: 768–778. 9101516

[26] Nicholas MK. The pain self-efficacy questionnaire: taking pain into account. Eur J Pain. 2007;11: 153–163. doi: 10.1016/j.ejpain.2005.12.008 16446108

[27] Adachi T, Nakae A, Maruo T, Shi K, Shibata M, Maeda L, et al. Validation of the Japanese version of the pain self-efficacy questionnaire in Japanese patients with chronic pain. Pain Med. 2014;15: 1405–1417. doi: 10.1111/pme.12446 24717053

[28] Carver CS. You want to measure coping but your protocol’s too long: consider the Brief COPE. Int J Behav Med. 1997;4: 92–100. doi: 10.1207/s15327558ijbm0401_6 16250744

[29] Baumstarck K, Alessandrini M, Hamidou Z, Auquier P, Leroy T, Boyer L. Assessment of coping: a new French four-factor structure of the Brief COPE inventory. Health Qual Life Outcomes. 2017;15: 8. doi: 10.1186/s12955-016-0581-9 28077139 PMC5225566

[30] Otsuka Y. The COPE Inventory: A theoretically based coping questionnaire. Hiroshima Psychol Res. 2008;8: 121–128.

[31] Psychology Foundation of Australia. Depression anxiety depression scales (DASS). 2014. Available from: http://www2.psy.unsw.edu.au/dass/.

[32] Henry JD, Crawford JR. The short-form version of the Depression Anxiety Stress Scales (DASS-21): construct validity and normative data in a large non-clinical sample. Br J Clin Psychol. 2005;44: 227–239. doi: 10.1348/014466505X29657 16004657

[33] Shelby RA, Somers TJ, Keefe FJ, DeVellis BM, Patterson C, Renner JB, et al. Brief Fear of Movement Scale for osteoarthritis. Arthritis Care Res (Hoboken). 2012;64: 862–871. doi: 10.1002/acr.21626 22290689 PMC3357444

[34] Kori S, Miller R, Todd D. Kinesiophobia: a new view of chronic pain behavior. Pain Manag. 1990;3: 35–43.

[35] Matsudaira K, Inuzuka K, Kikuchi N, Sagae C, Arisaka M, Isomura T, et al. Development of a Japanese version of the Tampa Scale for Kinesiophobia (TSK-J): translation and linguistic validation. Rinsho Seikei Geka. 2013;48: 13–19.

[36] Kikuchi N, Matsudaira K, Sawada T, Oka H. Psychometric properties of the Japanese version of the Tampa Scale for Kinesiophobia (TSK-J) in patients with whiplash neck injury pain and/or low back pain. J Orthop Sci. 2015;20: 985–992. doi: 10.1007/s00776-015-0751-3 26201395

[37] Sullivan MJL, Bishop SR, Pivik J. The Pain Catastrophizing Scale: development and validation. Psychol Assess. 1995;7: 524–532.

[38] Osman A, Barrios FX, Gutierrez PM, Kopper BA, Merrifield T, Grittmann L. The Pain Catastrophizing Scale: further psychometric evaluation with adult samples. J Behav Med. 2000;23: 351–365. doi: 10.1023/a:1005548801037 10984864

[39] Matsuoka H, Sakano Y. Assessment of cognitive aspect of pain: development, reliability, and validation of Japanese version of Pain Catastrophizing Scale. Jpn J Psychosom Med. 2007;47: 95–102.

[40] Bellamy N, Buchanan WW, Goldsmith CH, Campbell J, Stitt LW. Validation study of WOMAC: a health status instrument for measuring clinically important patient relevant outcomes to antirheumatic drug therapy in patients with osteoarthritis of the hip or knee. J Rheumatol. 1988;15: 1833–1840. 3068365

[41] Hashimoto H, Hanyu T, Sledge CB, Lingard EA. Validation of a Japanese patient-derived outcome scale for assessing total knee arthroplasty: comparison with Western Ontario and McMaster Universities osteoarthritis index (WOMAC). J Orthop Sci. 2003;8: 288–293. doi: 10.1007/s10776-002-0629-0 12768467

[42] Farrar JT, Young JP, LaMoreaux L, Werth JL, Poole MR. Clinical importance of changes in chronic pain intensity measured on an 11-point numerical pain rating scale. Pain. 2001;94: 149–158. doi: 10.1016/S0304-3959(01)00349-9 11690728

[43] Marx RG, Menezes A, Horovitz L, Jones EC, Warren RF. A comparison of two time intervals for test-retest reliability of health status instruments. J Clin Epidemiol. 2003;56: 730–735. doi: 10.1016/s0895-4356(03)00084-2 12954464

[44] Fayers PM, Machin D. Quality of life: the assessment, analysis and reporting of patient-reported outcomes. 3rd ed. West Sussex: Wiley-Blackwell; 2016.

[45] Landis JR, Koch GG. The measurement of observer agreement for categorical data. Biometrics. 1977;33: 159–174. 843571

[46] Boonstra AM, Stewart RE, Köke AJ, Oosterwijk RF, Swaan JL, Schreurs KM, et al. Cut-off points for mild, moderate, and severe pain on the numeric rating scale for pain in patients with chronic musculoskeletal pain: variability and influence of sex and catastrophizing. Front Psychol. 2016;7: 1466. doi: 10.3389/fpsyg.2016.01466 27746750 PMC5043012

[47] Lovibond SH, Lovibond PF. Manual for the depression anxiety stress scales. 2nd ed. Sydney: Psychology Foundation of Australia; 1995.

[48] Vlaeyen JWS, Kole-Snijders AMJ, Boeren RGB, van Eek H. Fear of movement/(re)injury in chronic low back pain and its relation to behavioral performance. Pain. 1995;62: 363–372. doi: 10.1016/0304-3959(94)00279-N 8657437

[49] Scott W, Wideman TH, Sullivan MJ. Clinically meaningful scores on pain catastrophizing before and after multidisciplinary rehabilitation: a prospective study of individuals with subacute pain after whiplash injury. Clin J Pain. 2014;30: 183–190. doi: 10.1097/AJP.0b013e31828eee6c 23552561

[50] Tighe CA, Youk A, Ibrahim SA, Weiner DK, Vina ER, Kwoh CK, et al. Pain catastrophizing and arthritis self-efficacy as mediators of sleep disturbance and osteoarthritis symptom severity. Pain Med. 2020;21: 501–510. doi: 10.1093/pm/pnz187 31504838 PMC8483157

